# The Success and Failure of the Schwann Cell Response to Nerve Injury

**DOI:** 10.3389/fncel.2019.00033

**Published:** 2019-02-11

**Authors:** Kristjan R. Jessen, Rhona Mirsky

**Affiliations:** Department of Cell and Developmental Biology, University College London, London, United Kingdom

**Keywords:** PNS, repair cell, nerve injury, regeneration, c-Jun, re-programming, Schwann cell

## Abstract

The remarkable plasticity of Schwann cells allows them to adopt the Remak (non-myelin) and myelin phenotypes, which are specialized to meet the needs of small and large diameter axons, and differ markedly from each other. It also enables Schwann cells initially to mount a strikingly adaptive response to nerve injury and to promote regeneration by converting to a repair-promoting phenotype. These repair cells activate a sequence of supportive functions that engineer myelin clearance, prevent neuronal death, and help axon growth and guidance. Eventually, this response runs out of steam, however, because in the long run the phenotype of repair cells is unstable and their survival is compromised. The re-programming of Remak and myelin cells to repair cells, together with the injury-induced switch of peripheral neurons to a growth mode, gives peripheral nerves their strong regenerative potential. But it remains a challenge to harness this potential and devise effective treatments that maintain the initial repair capacity of peripheral nerves for the extended periods typically required for nerve repair in humans.

## Introduction: Overview of Schwann Cells and Nerve Injury

Examination of the Schwann cells in uninjured nerves shows two surprisingly different cell types. One of them is the rather inconspicuous Remak cell, or non-myelin Schwann cell. These cells envelop all the small diameter axons, including many sensory axons and the all-important axons of the autonomic nervous system. The axons lie in troughs along the cell surface, and in rodents each cell generally ensheaths several axons, although in human nerves, one axon per Remak cell is common. The larger axons, including some sensory axons and the axons of motor neurons, are wrapped by myelin Schwann cells. They are 2–3 times longer that Remak cells and much bulkier, containing the myelin sheath, which is formed by the Schwann cell membrane wrapping multiple times around the axon and condensing to form a compact myelin cuff around the axon.

**Figure 1:**
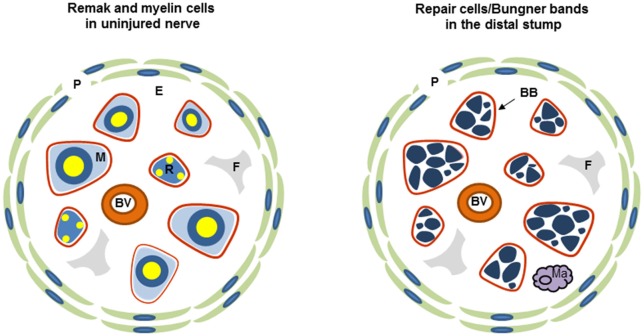
Diagrammatic representation of uninjured and injured nerve. Each diagram shows one fascicle and its main cellular constituents. Red line: basal lamina of Schwann cells (the basal lamina associated with perineurium and blood vessels is not shown), P, perineurium; R, Remak Schwann cell; M, myelin Schwann cell; F, fibroblast; E the connective tissue of the endoneurium, Ma, macrophage; BB, Bungner band containing transverse profiles of several repair cells and surrounded by a basal lamina.

Both Remak and myelin cells are coated by a basal lamina, outside of which lies the connective tissue, the endoneurium, which contains fibroblasts, blood vessels and a few macrophages and is ultimately surrounded by a multi-layered cellular tube, the perineurium (Figure 1). This assembly is termed a fascicle. Small nerves are uni-fascicular, while large nerves contain many fascicles bound together by connective tissue, the epineurium.

While both Schwann cells are thought to provide axons with metabolic and trophic support, only the myelin cells have the key role of accelerating nerve impulse conduction. Remak and myelin cells also express some common proteins, such as S100, a classical Schwann cell marker, while at the same time each cell possesses a characteristic molecular profile (reviewed in Jessen and Mirsky, 2005, 2016; Glenn and Talbot, 2013; Brosius Lutz and Barres, 2014; Monk et al., 2015). Thus, Remak cells express several markers also found on developing Schwann cells, such as neural cell adhesion molecule (NCAM), p75 neurotrophin receptor (p75NTR) and glial fibrillary acidic protein (GFAP), and L1 NCAM. The myelin cells on the other the hand express a large range of molecules that relate to the synthesis, maintenance or structure of the myelin sheath. This includes the major pro-myelin transcription factor Egr2 (Krox20), high levels of the enzymes that control cholesterol synthesis, structural proteins such as myelin protein zero (MPZ) and myelin basic protein (MBP), and membrane associated proteins like myelin associated glycoprotein (MAG), PMP22, and periaxin.

**Figure 2:**
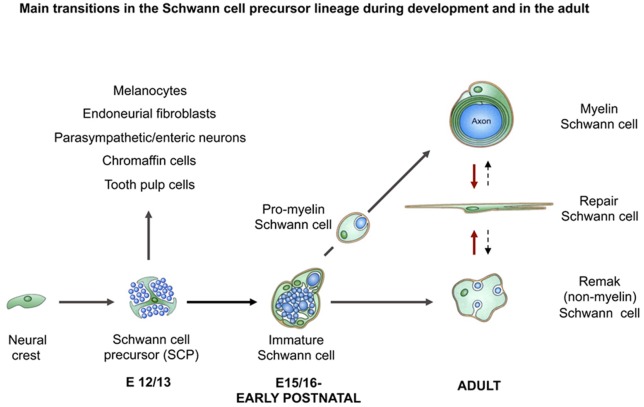
The main transitions in the Schwann cell lineage during development and after injury. Black uninterrupted arrows show normal development. Red arrows show the Schwann cell injury response. Black stippled arrows show post-repair reformation of Remak and myelin cells (with permission from Jessen et al., 2015b).

As different as Remak and myelin cells appear, these cells nevertheless originate developmentally from a common cell, the immature Schwann cell, present in rodent nerves in the perinatal period (Figure 2). These cells, in turn, derive from a distinct glial cell of embryonic nerves, the Schwann cell precursor. The conversion of Schwann cell precursors to Schwann cells is controlled by transcription factors such as AP2α, Zeb2, and Notch, and extracellular signals including endothelin and axon-associated neuregulin, which is essential for precursor survival (reviewed in Jessen and Mirsky, 2005; Jessen et al., 2015b; Quintes and Brinkmann, 2017).

Schwann cell precursors unambiguously exhibit a glial phenotype, expressing characteristic glial features, such as ensheathing axons within nerves and expressing Schwann cell associated mRNAs, such as those encoding for the major myelin protein MPZ and desert hedgehog (Dhh). It is interesting, however, that these cells also retain one notable feature of the neural crest cells from which they originate, namely they have a broad developmental potential. Thus, Schwann cell precursors give rise to cells such as melanocytes, endoneurial fibroblasts and neurons, in addition to Schwann cells, during embryonic development (reviewed in Jessen et al., 2015b; Kastriti and Adameyko, 2017).

One of the most interesting biological properties of Schwann cells is their plasticity (reviewed in Boerboom et al., 2017; Castelnovo et al., 2017; Jacob, 2017; Ma and Svaren, 2018). The phenotype adopted by Schwann cells, such as the Remak or myelin phenotypes described above, is strikingly dependent on signals in the cellular environment. Thus, all the evidence indicates that if a Remak cell was placed in contact with large diameter axon it would adopt the myelin phenotype and conversely, a myelin cell would convert to a Remak cell if associated with small diameter axons. This phenotypic instability may pre-dispose Schwann cells to demyelinating disease, since myelin Schwann cells may regress from myelin maintenance relatively easily in response to mutations that disturb cellular homeostasis. After nerve damage, Schwann cell plasticity and the ready response to environmental signals initially helps to provide nerves with strong regenerative potential, but at later times after injury, these features contribute to regenerative failure, as repair supportive Schwann cells are not sustained during the long times required for nerve repair in humans.

After nerve injury, the Schwann cells distal to the damaged area lose contact with axons as they degenerate. This represents a radical change in the signaling environment, because key signals for the control Schwann cell phenotype come from axons. Subsequently, further change is provided by a cocktail of bioactive factors secreted by macrophages, which invade injured nerves in large numbers. In response to these perturbations, Schwann cells show a remarkably fortunate response. This is the conversion of Remak and myelin cells, within their original basal lamina tubes, to a Schwann cell phenotype, the repair Schwann cell, which is specialized to encourage regeneration (reviewed in Jessen and Mirsky, 2016; Figure 2). These cells execute a repair program, a partly overlapping sequence of phenotypic changes involving myelin autophagy, expression of cytokines that call in macrophages for later stages of myelin clearance, activation of trophic factor expression and cellular elongation and branching to form regeneration tracks, called Bungner Bands. In this way, repair cells clear myelin, support the survival of injured neurons, axon regeneration and target innervation. These are the cells present in the distal stump of injured nerves along which regenerating axons navigate after injury, often for months or even years in humans due to the slow rate of axon growth.

One of the main problems in human nerve repair is that the phenotype of repair cells is not stable, but fades with time as the cells fail to maintain expression of trophic factors that promote axonal growth, likely due to gradual changes in the signaling environment within the chronically denervated distal stump (reviewed in Höke, 2006b; Sulaiman and Gordon, 2009). Furthermore, the repair cell population is not stable, their number eventually declining to very low levels. The reasons for the deterioration of repair cells are poorly understood, although the transcription factors STAT3 and c-Jun have been implicated in this process.

Importantly, PNS neurons also respond to axonal damage by activating an extensive gene program that facilitates axonal regeneration, a response classically referred to as the cell body response or the signaling to growth mode switch (Allodi et al., 2012; Blesch et al., 2012; reviewed in Fu and Gordon, 1997; Doron-Mandel et al., 2015).

Therefore, in response to damage, both neurons and Schwann cells convert to cell states that are specialized to deal with injury and promote healing. Comparable reprogramming of differentiated cells in response to injury can also be seen in other systems. This includes the conversion of fibroblasts to myofibroblasts during wound healing, and of pigmented epithelial cells to lens cells in the eye, the conversions of supportive cells to hair cells in the ear, hepatocytes to biliary epithelial cells in the liver, and of endocrine α to β cells in the pancreatic isles. In all of these cases, differentiated cells change identity in order to promote tissue homeostasis and repair. This type of change has therefore been termed adaptive cellular reprogramming (reviewed in Jessen et al., 2015a).

Eventually, axons that have successfully regenerated induce ensheathing repair cells to adopt again the Remak and myelin phenotypes, thereby restoring the nerve to its functional state. The repair Schwann cells are therefore a transient population, which exists only while they are needed.

We will now discuss some of the issues aired above in more detail.

## The Schwann Cell Injury Response: The Generation of Repair Cells

Unfortunately, in humans most nerve injuries involve nerve transection rather than the more easily repaired nerve crush. After a nerve crush, the basal lamina around each axon/Schwann cell unit remains intact and an axon stays within its basal lamina tube as it grows across the injury site to reach the distal stump. It therefore has a favorable possibility to reconnect with its original target tissue and restore function.

**Figure 3:**
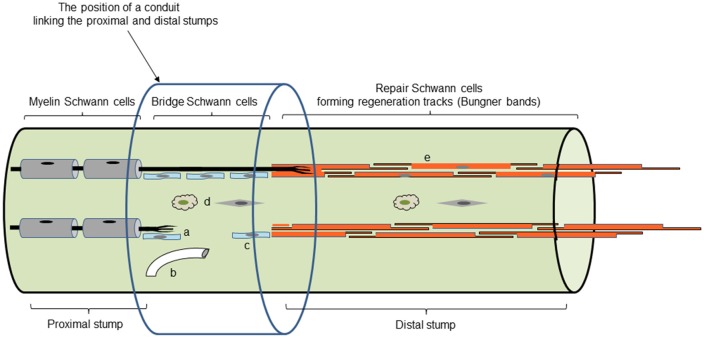
Main cell and tissue components of regenerating nerves. Repair cells in Bungner bands are shown in dark orange (e). Light blue shows the Schwann cells of the tissue bridge, some of which migrate out from the distal stump (c), while others accompany regenerating axons forming regeneration units (a). Not shown is the basal lamina that covers the Schwann cells in the proximal stump and the Bungner bands in the distal stump. The bridge Schwann cells receive important signals from blood vessels (b), fibroblasts and macrophages (d). The position of an inserted regeneration conduit is also shown (although the cellular events within it will depend on the nature of the conduit; with permission from Jessen and Arthur-Farraj, 2019).

In contrast, after cut, the connective tissue and basal lamina tubes are disrupted. Regeneration units consisting of axons accompanied by Schwann cells grow through a tissue bridge that forms between the proximal and distal nerve stumps. As a result of complex signaling between axons, Schwann cells and macrophages, involving factors such as Sox2, ephrin-B/EphB2 and TGFb, and guidance by fibroblasts and blood vessels, axons regenerate across the bridge to meet the Schwann cells that grow from the transected end of the distal stump (Parrinello et al., 2010; Cattin et al., 2015; Clements et al., 2017; reviewed in Cattin and Lloyd, 2016). Thus in cut nerves, continuity between the proximal stump and the target is broken, and axons are much less likely to find their way into their original basal lamina tubes and their target tissues, compromising proper restoration of function (Morris et al., 1972; Friede and Bischhausen, 1980; Meller, 1987; Barrette et al., 2008). The standard clinical treatment is to re-attach the proximal and distal nerve stumps. Although this leaves only a microscopic gap to be filled by a bridge, the problem of axons finding their original basal lamina tubes remains (Witzel et al., 2005). This problem is amplified when extensive injuries are repaired by the insertion of a nerve graft or an artificial insert (Figure 3). The interesting and intricate cellular interactions in the bridge region will not be discussed here in detail (for review see Cattin and Lloyd, 2016).

It does not matter whether axons are interrupted by crush or cut, in the distal stump the response of Schwann cells to injury is similar. In both cases, it converts Remak and myelin cells to repair supportive Schwann cells. The Schwann cell injury response has two principal components and will be discussed in terms of myelin Schwann cells only although similar principles are likely to apply to the response of Remak cells. The reversal of myelin differentiation represents one component. Genes coding for Egr2 (Krox20), cholesterol-related enzymes and the myelin-related proteins MPZ, MBP, MAG and periaxin are down-regulated. Conversely, molecules that characterize developing Schwann cells and adult Remak cells including, NCAM, p75NTR, GFAP and L1 are up-regulated (reviewed in Chen et al., 2007; Jessen and Mirsky, 2008; Martinez et al., 2015; Boerboom et al., 2017).

The second important part of the injury response is the appearance of a set of repair-supportive characteristics that are not seen, or seen in a muted form, in Schwann cells in normal mature nerves or in Schwann cells in developing nerves. This repair component of the response includes a number of elements (reviewed in Jessen and Mirsky, 2016).

1. Factors that promote the survival of injured neurons and axonal elongation are up-regulated. These include neurotrophic factors and surface molecules such as glial cell line-derived neurotrophic factor (GDNF), artemin, brain-derived neurotrophic factor (BDNF), neurotrophin-3 (NT3), nerve growth factor (NGF), vascular endothelial growth factor (VEGF), erythropoietin, FGFs, pleiotrophin, N-cadherin and p75NTR (Grothe et al., 2006; Fontana et al., 2012; Brushart et al., 2013; reviewed in Boyd and Gordon, 2003; Chen et al., 2007; Scheib and Höke, 2013; Wood and Mackinnon, 2015).
2. An innate immune response is activated. This involves the upregulation of cytokines including tumor necrosis factor α (TNFα), interleukin-1α (Il-1α), Il-1β, leukemia inhibitory factor (LIF), monocyte chemotactic protein-1 (MCP-1) and toll-like receptors by the Schwann cells in the distal stump (reviewed in Martini et al., 2008; Rotshenker, 2011). This allows repair Schwann cells to recruit macrophages and other immune system cells such as neutrophils to the nerve, promoting nerve regeneration in several ways. Cytokines including Il-6 and LIF attract macrophages to the nerve but also act directly on neurons to promote axon growth (Hirota et al., 1996; Cafferty et al., 2001; reviewed in Bauer et al., 2007). An additional sustained source of cytokines are macrophages that invade nerves and ganglia. They also promote vascularization of the nerve bridge between the proximal and distal stumps (Barrette et al., 2008; Niemi et al., 2013; Cattin et al., 2015). Macrophages also co-operate with Schwann cells to degrade myelin debris (see further below; reviewed in Hirata and Kawabuchi, 2002; Rotshenker, 2011).
3. Schwann cells proliferate and then undergo a striking about three-fold elongation as they form the regeneration tracks, Bungner bands, which are essential for guiding axons back to their target areas (see further below). These structural changes are a component of the tissue remodeling which transforms the distal stump into a collection of regeneration tracks (Gomez-Sanchez et al., 2017).
4. Repair cells activate mTOR-independent autophagy, myelinophagy, to break down their myelin sheaths, which are redundant after axonal degeneration (Gomez-Sanchez et al., 2015; Suzuki et al., 2015; Jang et al., 2016; Brosius Lutz et al., 2017).

## The Properties of Repair Cells

### Repair Schwann Cells Show a Distinct Molecular Profile

At least two genes, *Olig 1, Shh*, differentiate repair cells from myelin and Remak cells, and also from immature Schwann cells, and Schwann cell precursors in embryonic nerves. These genes are highly up-regulated by c-Jun in Schwann cells of injured nerves. These genes are distinctive markers of repair Schwann cells because they are expressed de novo after injury This applies also to *GDNF*, with the exception that this gene is detected in embryonic Schwann cell precursors, although it is down-regulated before birth (Lu et al., 2000; Zhou et al., 2000; Piirsoo et al., 2010; Arthur-Farraj et al., 2012; Fontana et al., 2012; Lin et al., 2015).

In addition, a gene expression screen found over a hundred genes that were significantly up- or down-regulated in the distal stump after damage, although they were not regulated during development, since they were present at similar levels in developing and adult uninjured nerves. The majority of these genes were up-regulated by injury. This reveals a substantial group of genes that are specifically activated in injured nerves, and which are therefore candidate markers for repair cells (Bosse et al., 2006).

### Timing and the Repair Program

The different components of the repair program are not switched on synchronously. Instead, each of them reaches peak expression at different times after injury. For instance, protein levels of cytokines such as Il-1β and TNFα, peak within 1 day of injury, but are sharply lower at 3 days (Rotshenker, 2011), autophagy is high at 5 days and reduced thereafter (Gomez-Sanchez et al., 2015), GDNF protein levels peak at about 1 week after injury and BDNF is maximally expressed after 2–3 weeks (Eggers et al., 2010). The transcription factor c-Jun which is an important regulator of the repair program (see below), is activated within hours of injury, but c-Jun protein levels continue to increase for at least 7–10 days and cellular elongation continues for at least 4 weeks after injury (Gomez-Sanchez et al., 2017). The repair program therefore represents a temporal sequence of events that overlap and co-operate to support repair.

### The Elongation and Branching of Repair Cells

**Figure 4:**
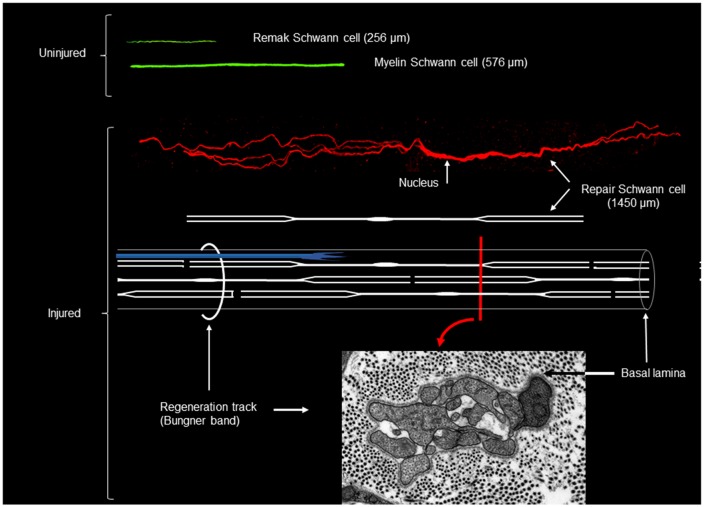
Myelin, Remak and repair Schwann cells after genetic labeling *in vivo*. Shown in green are myelin and Remak cells of average length as they appear in uninjured nerve. In red is an example of a long, branched repair cell (generated from a myelin cell), in 4 week cut nerve without reinnervation. All cells are shown to scale. Shown also is a schematic diagram of a repair cell and the assembly of these cells to form a Bungner band enclosed by a basal lamina and containing a regenerating axon (in blue). The electron micrograph shows a transverse section of a Bungner band (red image printed with permission from Gomez-Sanchez et al., 2017). Scale bar: 1 μm.

One of the cardinal features of Schwann cells is their extended morphology, a feature that is often associated with the requirement to stretch out to cover the elongated axons. However, Schwann cells which lose contact with degenerating axons in the distal stump do not shorten. Rather, lineage tracing studies have shown that loss of axonal contact triggers a striking cellular elongation (Gomez-Sanchez et al., 2017). One week after nerve transection without regeneration, Remak cells have doubled in length, and 4 weeks after injury they are three-fold longer than Remak cells in intact nerves. Similarly, myelin cells have doubled in length by 4 weeks after injury. Loss of axonal contact also induces the cells to branch. About 50% and 30% of repair cells derived from Remak and myelin cells, respectively, form branches, which are often long and lie parallel to the main axis of the cell (Gomez-Sanchez et al., 2017; Figure 4).

This study also addressed two long-standing assumptions about Schwann cells. First, that Remak and myelin cells generate the cells found in the Bungner bands of injured nerves, and second, that the Bungner band cells, in turn, generate the myelin cells found in regenerated nerves. These fundamental presumptions were confirmed directly using linage tracing methods (Gomez-Sanchez et al., 2017). This allowed repair cells from Remak cells and myelin cells to be studied separately, and made it possible to identify the progeny of myelin cell-derived repair cells among the cells that re-myelinate nerves after regeneration. This revealed that re-myelination involves a remarkable about seven-fold cell shortening, as the elongated repair cells wrap axons to generate the typically short myelin internodes of regenerated nerves.

Although Remak and myelin cells have sharply different structure, they generate repair cells with broadly similar morphology. The extremely elongated and branched structure of these cells differentiates repair cells from other cells in the Schwann cell lineage. This morphology also promotes the generation of uninterrupted and robust regeneration tracks by maximizing cell-cell overlap within each Bungner band.

### Myelin Clearance

Unlike the CNS, peripheral nerves are able to clear redundant myelin after axonal degeneration, a feature that is widely believed to facilitate regeneration because of the growth-inhibitory nature of myelin (Kang and Lichtman, 2013; reviewed in Hirata and Kawabuchi, 2002; Rotshenker, 2011). Nerves clear myelin by two distinct mechanisms. First, Schwann cells switch from myelin maintenance to actively breaking down their own myelin sheaths through the mechanism of myelin autophagy (Gomez-Sanchez et al., 2015; Suzuki et al., 2015; Jang et al., 2016; Brosius Lutz et al., 2017). This process delivers myelin to Schwann cell lysosomes for degradation, following internalization of myelin in the form of myelin ovoids (Jung et al., 2011), and the formation of smaller cytoplasmic myelin fragments. About 50% of total myelin is thought to be broken down by Schwann cells (Perry et al., 1995). Second, myelin debris is phagocytosed and degraded Schwann cells and in particular by macrophages, which gradually invade injured nerves, recruited by the repair cell cytokine expression referred to above.

## Control of Repair Cell Generation

### Repair Schwann Cells Are Controlled by Dedicated Signaling Mechanisms

A number of mechanisms take part in regulating the Schwann cell injury response. Importantly, it has become clear that this includes regulatory mechanisms that operate selectively in repair cells, and have only a minor or undetectable function in developing Schwann cells. This applies to the transcription factors c-Jun and STAT3, which will be discussed in subsequent sections. Regulation of histone H3K27 trimethylation represents another selective mechanism, which appears not to be significantly involved in control of Schwann cell development (Ma et al., 2016). In response to injury, however, the activation of several injury-related genes is promoted by removal of the repressive histone mark H3K27 trimethylation at their promoter regions, combined with the gain of the active histone mark H3K4 methylation. At the same time, the active histone mark H3K27 acetylation is lost from the enhancers of myelin genes as their expression fades (Hung et al., 2015; Ma et al., 2016). An important, selective role in repair cells is also played by the tumor suppressor protein Merlin. Schwann cell specific inactivation of Merlin has major adverse effects on repair cell function, axonal regeneration and re-myelination, but only minor effects on Schwann cell development (Mindos et al., 2017). Together with the c-Jun and STAT3, these histone methylation events described above, and merlin, represent gene regulatory mechanisms, which selectively control repair cells, and appear unimportant in developing Schwann cells.

### Other Signals That Control the Schwann Cell Injury Response

Positive regulators of repair cell generation or function include Notch (Woodhoo et al., 2009), Sox2 (Parrinello et al., 2010; Roberts et al., 2017), gpr126 (Mogha et al., 2016), TGFb (Morgan et al., 1994; Stewart et al., 1995; Cattin et al., 2015; Clements et al., 2017) and ERK1/2, although its function is complex, since it ERK1/2 activation is also required for myelin synthesis (Harrisingh et al., 2004; Fischer et al., 2008; Newbern et al., 2011; Napoli et al., 2012; Sheean et al., 2014; Cervellini et al., 2018; reviewed in Monk et al., 2015; Jessen and Mirsky, 2016; Boerboom et al., 2017). The transcription factor Zeb2 is required for normal re-myelination after injury, but appears not to be important for the generation of repair cells (Quintes et al., 2016; Wu et al., 2016). Toll-like receptors are involved in up-regulation of cytokines, macrophage recruitment and myelin clearance after injury, while the mTORC1 pathway is also involved in myelin clearance and is needed for effective expression of c-Jun and other repair cell genes after injury (Boivin et al., 2007; Norrmén et al., 2018). The significance of these proteins for axonal regeneration is not clear.

Negative regulation of repair cells is exerted by histone deacetylase 2 (HDAC2), which is activated after injury acts to suppress c-Jun and delay the generation of repair cells. Inhibition of HDAC2 therefore represents a potential route to improve regeneration, because regeneration is accelerated when HDAC2 is inactivated, although re-myelination is impaired (Brügger et al., 2017; Jacob, 2017).

Considerable attention has been paid to the potential role of neuregulin in nerve injury. Schwann cells in transected nerves elevate expression of ErbB2/3 receptors and neuregulin-1 I/ll isoforms (Carroll et al., 1997; Stassart et al., 2013; Ronchi et al., 2016). Surprisingly, however, neuregulin-1 is not involved in injury-induced Schwann cell proliferation, and macrophage recruitment, and myelin breakdown appears normal in nerves without neuregulin (Atanasoski et al., 2006; Fricker et al., 2013). Another unexpected finding is that even after removal of neuregulin-1 from both Schwann cells and axons, re-myelination of regenerated nerves after injury is eventually normal after a substantial delay (Fricker et al., 2013; Stassart et al., 2013). This contrasts with development, since neuregulin-1, expressed by axons, is necessary for myelination by developing Schwann cells (Birchmeier and Nave, 2008). In another deviation form development, repair Schwann cell-derived neuregulin-1 has a significant role in accelerating re-myelination after injury, most likely working through an autocrine signaling loop, while Schwann cell-derived neuregulin-1 is not important for developmental myelination (Stassart et al., 2013). The involvement of neuregulin-1 in the control of myelination therefore differs between repair Schwann cells and developing cells.

Inactivation of neuregulin-1 in axons and Schwann cells results in slow axonal regeneration after injury. This suggests that endogenous neuregulin signaling through ErbB2/3 receptors in repair cells promotes the repair phenotype and enhances the capacity of these cells to support axon growth, but direct evidence for this mechanism of action is missing. Nevertheless, pharmacologically enhancing neuregulin signaling might serve as a tool for promoting nerve repair, because enforced Schwann cell ErbB2 expression, and exogenously applied neuregulin increase axonal regeneration *in vivo* (Ronchi et al., 2013; Han et al., 2017; reviewed in Gambarotta et al., 2013).

### The Function of c-Jun in Repair Cells

The transcription factor c-Jun plays a crucial role in the Schwann cell injury response (Jessen and Mirsky, 2016). c-Jun levels are low in uninjured nerves, but are rapidly and strongly elevated by injury (De Felipe and Hunt, 1994; Shy et al., 1996). When this is prevented, by selective inactivation of c-Jun in Schwann cells in transgenic mice (c-Jun cKO mice) regeneration of axons and recovery of function after injury are strikingly compromised. Uninjured nerves in these mice are essentially normal. This indicates that c-Jun is not essential for Schwann cell development, and that the role of this transcription actor is restricted to controlling the response of Schwann cells to nerve damage (Arthur-Farraj et al., 2012).

**Figure 5:**
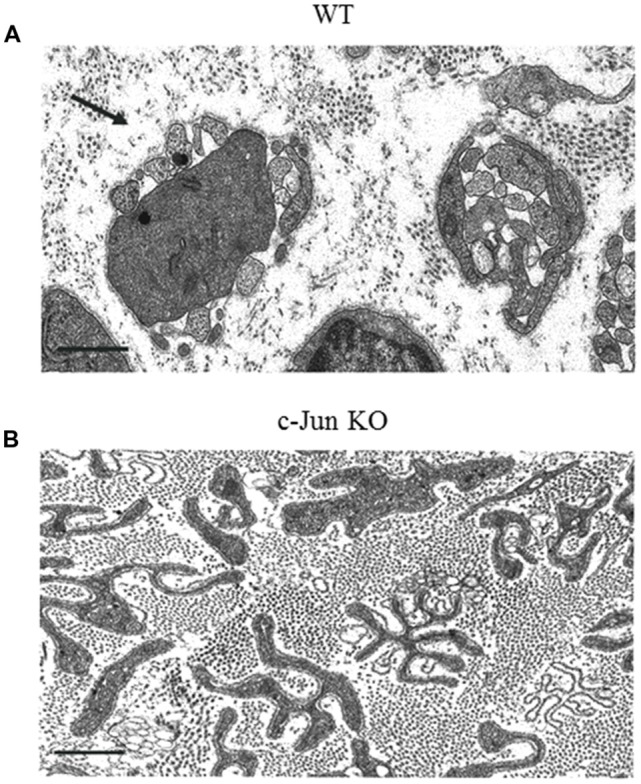
The structure of regeneration tracks (Bungner Bands) is controlled by Schwann cell c-Jun. Electron micrographs from the distal stump of mouse sciatic nerve 4 weeks after transection (without regeneration). **(A)** WT nerve showing classic regeneration tracks (Bands of Bungner; an example is arrowed). **(B)** Distorted regeneration tracks in c-Jun cKO nerve, containing irregular and flattened cellular profiles (with permission from Arthur-Farraj et al., 2012). Scale bar: 1 μm.

The regeneration failure in c-Jun cKO mice is due to the important function of c-Jun in injury-induced Schwann cell reprogramming. c-Jun directly or indirectly affects the expression levels of at least 172 genes of the ~4,000 genes that change expression in Schwann cells after injury. This gives c-Jun significant control over both parts of the Schwann cell injury response, de-differentiation of myelin cells and activation of the repair program (Arthur-Farraj et al., 2012, 2017). c-Jun helps de-differentiation, because it is needed for the normal down-regulation of myelin genes after injury. Among these are the genes encoding the transcription factor *Egr2 (Krox20)*, a master regulator of the myelin program, and the *MPZ* and *MBP* genes. The negative gene regulation by c-Jun and its cross-antagonistic relationship with Egr2 (Krox20) had been studies before its importance for regeneration was revealed and helped give rise to the idea that c-Jun, in combination with a group of other transcriptional regulators, including Notch, Sox2, Id2 and Pax3, functioned as negative regulators of myelination (Kioussi et al., 1995; Parkinson et al., 2004, 2008; Le et al., 2005; Doddrell et al., 2012; Fazal et al., 2017; Florio et al., 2018; reviewed in Jessen and Mirsky, 2008). Although these genes may be important for modifying the rate or onset of myelination in developing nerves, a key role for c-Jun-mediated gene down-regulation *in vivo* appears to be that of helping to suppress myelin gene expression in adult nerves after injury.

c-Jun also promotes the normal activation of the repair program, which it controls in several important ways (Arthur-Farraj et al., 2012; Fontana et al., 2012). First, in the absence of Schwann cell c-Jun (c-Jun cKO mice), important trophic factors and cell surface proteins that support survival and axon growth fail to be normally upregulated. This includes GDNF, artemin and BDNF, p75NTR and N-cadherin. Two of these, GDNF and artemin, have been shown to be direct c-Jun targets and have been implicated in sensory neuron death after injury (Fontana et al., 2012). Normally some dorsal root ganglion (DRG) sensory neurons and facial motoneurons die after sciatic and facial nerve injury, respectively, and in humans DRG neuron death is considered a major reason for poor outcomes of nerve regeneration (Faroni et al., 2015). Death of DRG neurons and facial motoneurons is greatly increased in c-Jun cKO mice. This shows that a key function for repair Schwann cells and c-Jun signaling is to support the survival of injured neurons. Second, the regeneration tracks formed by denervated Schwann cells without c-Jun have a disorganized structure (Figure 5). In culture, c-Jun is needed for the typical narrow, bi/tripolar Schwann cell morphology, since c-Jun-negative cells tend to be flat and sheet-forming. Similarly, *in vivo*, the repair Schwann cells within the regeneration tracks show grossly abnormal morphology when viewed in transverse electron micrograph sections. c-Jun appears to be necessary for the conversion of the complex and sheath-like structure of the myelin cells to the narrow, rod-like and branched structure of repair cells that is required for the formation of normal regeneration tracks. Third, c-Jun promotes myelinophagy, and c-Jun cKO nerves show a substantial delay in breakdown of myelin (Arthur-Farraj et al., 2012; Gomez-Sanchez et al., 2017).

Thus, c-Jun exerts a broad control over the Schwann cell injury response, and thereby neuronal survival and regeneration. It is therefore of interest that in two important situations where repair cells fail, namely chronic denervation and aging, c-Jun levels in these cells are reduced. This is discussed in “Nerve Injury Activates Epithelial Mesenchymal Transition (EMT) Genes” section below.

## Nerve Injury Activates Epithelial Mesenchymal Transition (EMT) Genes

Recent gene screen studies suggest that the generation of repair cells involves the activation of a process related to epithelial mesenchymal transitions (EMTs; Arthur-Farraj et al., 2017; Clements et al., 2017). RNA-Seq of distal nerve stumps 7 days after injury show enrichment for EMT mRNAs and miRNAs. This includes down-regulation of RNAs that are linked to mesenchymal-epithelial transition (MET), and up-regulation of RNAs associated with EMT. Enrichment for EMT genes was also shown in more detailed experiments using FACs to sort td-tomato labeled Schwann cells from injured nerves (Clements et al., 2017). This study also showed that EMT activation was strongest in the Schwann cells the tissue bridge, where arguably tissue remodeling is more radical than in the distal stump.

Typically, EMT-like processes involve also an increase in stemness, namely the activation of genes associated with stem cells (Fabregat et al., 2016; Liao and Yang, 2017). This is also seen in injured nerves, where the generation of repair cells is accompanied by activation of the Myc stemness and Core pluripotency modules and suppression of polycomb-related factors (Clements et al., 2017).

In many tissues, the combined activation of EMT and stemness is associated with increased cellular motility, proliferation, morphological flexibility, tissue remodeling, and the loosening up of differentiation states (Nieto et al., 2016; Forte et al., 2017). These events are key features of the response of many tissues to injury. Not surprisingly, therefore, the activation of EMT and stemness programs have now been established as a key physiological response to injury in various tissues.

The demonstration of EMT/stemness activation in Schwann cells of injured nerves brings the nerve injury response in line with injury responses in many other systems. It adds an important new component to our understanding of the injury response. In particular, the loosening of differentiation states associated with increased stemness is consistent with the notion that nerve injury triggers a shift in Schwann cell differentiation state from myelin and Remak phenotypes to a Schwann cell state specialized to promote repair.

## Why Does Regeneration Fail?

In the light of the radical, adaptive injury response of neurons and Schwann cells, which in many types of rodent experiments results in effective nerve repair, why is it that the clinical outcome after nerve injury in humans is generally poor? This paradox of PNS repair can be explained by a number of factors, including the hindrance to axon growth provided by the injury site and the difficulty of getting large numbers of axons from the proximal to the distal stump, misrouting of axons resulting in re-innervation errors, and the presence of growth-inhibiting extracellular matrix. Perhaps the most difficult problems arise from the relatively slow growth of axons. Because of this, most human nerve injuries involve chronic denervation of the more distal parts of damaged nerves and of the target tissues such as muscle. This results in target atrophy, while neurons gradually die and surviving neurons may fail to sustain their capacity to regenerate axons during the extended period required for repair (Höke, 2006a; Sulaiman and Gordon, 2013; Patel et al., 2018).

Here, we will consider another major issue in regeneration failure. This is the fact that the nerve stump distal to transection gradually loses the ability to support the growth of regenerating axons during chronic denervation. This has been shown in a number of studies, most of which represent cross-suturing, typically involving the suturing of acutely transacted tibial nerve to the distal stump of the common peroneal nerve, which had be transected either acutely, the control condition, or at different times up to 6 months previously. Other experiments for addressing the same question have studied regeneration through nerve grafts, where the graft is obtained from nerves that have been denervated previously for various lengths of time. All of the experiments agree that transected nerve stumps retain full or only mildly reduced capacity for supporting regeneration for about 1 month (Li et al., 1997; Sulaiman and Gordon, 2000; Kou et al., 2011; Jonsson et al., 2013). By 2 months, regeneration support remains unchanged in some studies (Li et al., 1997), although other reports show it reduced by about 40%–50% (Sulaiman and Gordon, 2000; Kou et al., 2011). At 3 months regeneration support is further diminished and reduced to very low levels by 6 months (Li et al., 1997; Sulaiman and Gordon, 2002; Gordon et al., 2011; Jonsson et al., 2013; see however Rönkkö et al., 2011).

While these experiments were carried out on rats, we find that also in mice, the capacity of distal stumps to support regeneration is reduced by about 50% after 2.5 months of chronic denervation (Wagstaff et al., 2017).

## Is the Failure of Distal Stumps to Support Growth Due to Lack of Schwann Cells?

The deterioration in the capacity of chronically denervated stumps to support regeneration could result from two separate factors: gradual reduction in Schwann cell numbers, or the fading of the repair phenotype of cells still present in the nerve. Both of these factors could in turn be influenced by signals from macrophages that invade injured nerves and are initially present in large numbers that decline with time. The question of whether Schwann cell number is a key factor raises two additional ones. First, what is known about Schwann cell numbers in chronically transacted nerves? Second, are variations in Schwann cell numbers within the range seen in cut nerves likely to affect regeneration?

While it is long established that nerve injury triggers a wave of Schwann cell proliferation, there is surprisingly little quantitative information on Schwann cell numbers in cut nerves after injury. Cell counts using electron microscopy show that 2 weeks after cut the number of Schwann cells in mouse sciatic nerve has risen about 2.5-fold compared to uninjured nerves and remains similar at 1 and 1.5 months (Wagstaff et al., 2017), Using S100 to identify Schwann cells in the cut sciatic nerve of rats show 3–4-fold increase at 1–2 weeks with little change at 1.5 months (Siironen et al., 1994). These workers also report a sharp drop between 1.5 and 2 months, but since S100 is present at reduced levels after injury, these figures might overestimate the reduction in Schwann cell number (Siironen et al., 1994). Another study also using S100 indicates that Schwann cell numbers at 2.5 months remains 2–3-fold that in uncut nerves (Salonen et al., 1988). Our data from mouse nerves show a drop of about 30% between 2 weeks and 2.5 months (Wagstaff et al., 2017).

A study employing a more indirect method involving counting of the number of cells obtained by enzymatic dissociation of nerves at different times after nerve cut without innervation, concluded that the number of Schwann cells drops by about 30% between 1 and 2 months with little change at 3 and 4 months (Li et al., 1998). The number of cells that can be isolated from nerves after 6 months of chronic denervation is only 10%–15% of that obtained at 4 weeks (Li et al., 1998; Jonsson et al., 2013).

Although the data are limited and not always consistent, the above suggests that 2–3 months after chronic denervation, a time when growth support by the distal stump is significantly reduced, the number of Schwann cells has dropped at most by about 30%–50% from the high cell number seen 1–4 weeks after injury. This would mean that the number of cells at 2–3 months remains substantially higher than the number in uninjured nerves.

Is it likely that this fall from peak cell numbers accounts for the reduction in regenerative support during chronic denervation? The answer to this question depends on the weight is put on the reports discussed below that imply that regeneration remains normal in nerves where injury-induced increase in Schwann cell numbers is prevented, and cell numbers in the distal stump therefore remain similar to those in uninjured nerves.

## Does Nerve Regeneration Depend on Schwann Cell Proliferation?

It is a common view that Schwann cell proliferation and expansion in Schwann cell numbers in injured nerves is required for regeneration (Hall and Gregson, 1977; Hall, 2005). Recent genetic approaches to specifically inhibit Schwann cell proliferation suggest, however, that this may not be the case. Injury-induced Schwann cell proliferation depends on cyclin D1, although this protein is not important for proliferation during development. Nerves in D1^−/−^ mice therefore develop normally, but Schwann cell proliferation after injury is blocked. Perhaps surprisingly, regeneration appears not to markedly affected in these mice (Kim et al., 2000; Atanasoski et al., 2001; Yang et al., 2008).

## Deterioration of Repair Cells: An Important Reason for Regeneration Failures

Since Schwann cell numbers after 2–3 months of chronic denervation are likely to remain at least double that in uninjured nerves, and in view of the experiments on cyclin1^−/−^ mice outlined above, it seems that lack of Schwann cells is unlikely to be the major reason for the reduction in growth support provided by 2–3 month chronically denervated stumps, although at later times ongoing reduction in cell number will eventually be significant. This suggests that the deterioration of repair cells is a two-stage process involving, first, surviving cells gradually down-regulating their repair phenotype, followed by death of cells that have by that time lost most of their regeneration-supportive properties. There is in fact ample evidence for such fading of the repair cell phenotype, manifest in reduced expression of neuron-supportive trophic factors during chronic denervation, including GDNF, BDNF, NT3 and NGF (Eggers et al., 2010; reviewed in Boyd and Gordon, 2003; Höke and Brushart, 2010).

Thus, the fading of the repair phenotype, combined in the long run with dwindling cell numbers, are likely to be the twin reasons for reduced growth support provided by chronically denervated stumps. It is therefore important to identify factors that control the long-term maintenance of repair cells, sustain the expression of repair supportive genes and support cell survival.

## Signals That Maintain Repair Cells

While there are as yet surprisingly few studies on this important topic, two transcription factors, STAT3 and c-Jun have recently been shown to have a role in repair cell maintenance (Benito et al., 2017; Wagstaff et al., 2017). Activation of Schwann cell STAT3 is triggered by injury, and largely sustained in long-term denervated repair cells (Sheu et al., 2000). Genetic inactivation of STAT3 in these cells results in decreased autocrine Schwann cell survival signaling and a striking loss of Schwann cells from chronically denervated stumps. Loss of STAT3 also reduces the expression of repair cell markers, including GDNF, BDNF and Shh, during long-term denervation (Benito et al., 2017). Inactivation of Schwann cell STAT3 does, however, not significantly affect nerve development. The important role of STAT3, therefore, is to acts as a brake on the deterioration of repair cells in chronically denervated adult nerves.

Unlike STAT3, c-Jun levels in Schwann cells decline significantly during long-term denervation, in tandem with the fading of the repair phenotype, reduced cell numbers, and reduced capacity to support regeneration. Significantly, the ability of long-term denervated repair cells to support regeneration can be restored to control levels by genetically enhancing c-Jun expression in these cells to levels similar to those in short-term denervated nerve stumps (Fazal et al., 2017; Wagstaff et al., 2017). This raises the possibility that the deterioration of chronically denervated repair cells is to a significant extent caused by a failure to sustain high c-Jun levels.

Aging, like chronic denervation, is accompanied by a marked reduction in the rate of nerve regeneration (Verdú et al., 2000; Painter, 2017). Using rodent models, this has been traced primarily to age-related deterioration of repair Schwann cells, since repair cells from older animals show reduced expression of repair cell markers and reduced ability to support axon growth (Painter et al., 2014). Many of the abnormally expressed genes are c-Jun regulated, and c-Jun activation after injury is also significantly reduced in older animals. These observations have implicated dysregulation of Schwann cell c-Jun in the age-related deterioration of nerve repair (Painter et al., 2014; Wagstaff et al., 2017). A support for this suggestion comes from experiments on transgenic animals in which c-Jun expression in injured old nerves has been elevated to levels similar to those of injured young nerves. This correction of Schwann cell c-Jun levels is sufficient to correct the age-related regeneration deficit, and accelerate the rate of axonal regeneration to that seen in young animals (Wagstaff et al., 2017). Therefore, the inability of repair Schwann cells in older animals to elevate c-Jun to high levels may be a significant factor in the age-related failure of nerve repair.

## Conclusions

The key driver of nerve regeneration, apart from the neurons themselves, are the denervated Schwann cells that make up the regeneration tracks, Bungner bands, that occupy nerves distal to the injury site. These repair Schwann cells are adapted to meet the particular needs that arise in injured adult nerves. They differ from other Schwann cells, having separate transcriptional and epigenetic mechanisms, distinct morphology and molecular profile, in addition to carrying out a set of functions, comprising the repair program, which support nerve regeneration.

The realization that these cells possess distinct properties and cell-type specific control mechanisms is a useful step forward. It will help to focus future work on learning how to manipulate these particular cells, how to amplify their repair-supportive functions, and how prevent their deterioration in older age and during the prolonged periods required for axonal regeneration in human nerves.

## Author Contributions

Both authors contributed equally to the writing and editing of the manuscript.

## Conflict of Interest Statement

The authors declare that the research was conducted in the absence of any commercial or financial relationships that could be construed as a potential conflict of interest.
